# Delivery Practices of Traditional Birth Attendants in Dhaka Slums, Bangladesh

**Published:** 2007-12

**Authors:** N. Fronczak, S.E. Arifeen, A.C. Moran, L.E. Caulfield, A.H. Baqui

**Affiliations:** 1 Social Sectors Development Strategies, Inc., 1411 Washington Street, Suite 6, Boston, MA 02118, USA; 2 ICDDR,B, GPO Box 128, Dhaka 1000, Bangladesh; 3 Department of International Health, Johns Hopkins Bloomberg Schoolof Public Health, 615 North Wolfe Street, Baltimore, MD 21205, USA

**Keywords:** Birth practices, Community studies, Delivery, Maternal health, Postpartum morbidity, Prospective studies, Traditional birth attendants, Bangladesh

## Abstract

This paper describes associations among delivery-location, training of birth attendants, birthing practices, and early postpartum morbidity in women in slum areas of Dhaka, Bangladesh. During November 1993–May 1995, data on delivery-location, training of birth attendants, birthing practices, delivery-related complications, and postpartum morbidity were collected through interviews with 1,506 women, 489 home-based birth attendants, and audits in 20 facilities where the women from this study gave birth. Associations among maternal characteristics, birth practices, delivery-location, and early postpartum morbidity were specifically explored. Self-reported postpartum morbidity was associated with maternal characteristics, delivery-related complications, and some birthing practices. *Dais* with more experience were more likely to use potentially-harmful birthing practices which increased the risk of postpartum morbidity among women with births at home. Postpartum morbidity did not differ by birth-location. Safe motherhood programmes must develop effective strategies to discourage potentially-harmful home-based delivery practices demonstrated to contribute to morbidity.

## INTRODUCTION

Postpartum morbidity is common among women in Bangladesh. A national survey showed that 24% of women reported at least one complication during the postpartum period (1), while two studies in urban slum areas of Dhaka, Bangladesh, demonstrated that approximately 75% of women reported at least one postpartum morbidity (2,3). The World Health Organization (WHO) currently recommends that all births are assisted by a skilled attendant to address unacceptably high levels of maternal mortality and morbidity (4). In Bangladesh, although women living in urban slum areas of Dhaka reside in close proximity to facilities with skilled care, 70% of women in urban areas give birth at home with non-medically trained providers (5) which is likely to be even higher in urban slums.

Postpartum morbidity can be attributed to (a) maternal heath status prior to pregnancy; (b) conditions which develop during pregnancy; and (c) complications or conditions which occur as a result of childbirth. Other factors that influence postpartum morbidity include maternal traits, such as primiparity, grand-multiparity, and short stature, and socioeconomic factors, such as poverty, access to care, and low education (6–11).

The level of training of birth attendants and the management of complications by both home and facility-based attendants can contribute to postpartum morbidity (7,9–11). Harmful practices for childbirth include: giving birth on a dirty surface; lack of hand-washing by the birth attendant; guarding the perineum with the foot; frequent vaginal examinations; and traditional methods commonly used to stop bleeding, such as pressure on the abdomen with hand, knee, stool, or other objects, and methods to hasten delivery of the infant or to expel the placenta (12–16). Other delivery practices, such as using oxytocic drugs to augment labour, internal version to re-position malpositioned infants, and manually removing the placenta, are considered unsafe if used by untrained persons (12–16).

Previously, a high incidence of self-reported delivery-related complications was documented among 1,506 women living in urban Dhaka (2). In this paper, the types of delivery-care providers used by these women, their training and experience, and reported birthing practices are described. Specifically, the associations among place of delivery, training and experience of home-delivery providers, childbirth practices, and postpartum morbidity were explored.

## MATERIALS AND METHODS

This community-based prospective study was conducted in 424 clusters of 25-50 households in five selected low-income upazilas (administrative areas) of Dhaka city, the capital of Bangladesh. Slum clusters were chosen using a multistage probability-sampling methodology. Women who completed at least seven months of pregnancy and who planned to give birth in Dhaka were eligible for the study. During November 1993–May 1995, trained data collectors interviewed pregnant women in their homes in these households (n=1,506) at seven months gestation and at 72 hours and seven days postpartum. At 14-22 days postpartum, trained female physicians interviewed and examined women. In these interviews, information on economic status, prior history of pregnancy, self-reported complications, childbirth experience, place of delivery, and self-reported postpartum morbidities was collected. Information collected previously at 72 hours and 14-22 days postpartum was used for categorizing self-reports of delivery-related complications and subsequent postpartum morbidity using operational definitions (2). Here, this morbidity information is linked to the labour and delivery experience reported by the mother at 72 hours postpartum. Home-based birth attendants identified by study women were interviewed regarding formal training and experience in conducting deliveries. Data were also collected from 20 facilities where the study women delivered, including equipment, supplies, and clinical records of complications and morbidity.

The interview at 72 hours postpartum began with open-ended questions to elicit a narrative description of the woman's delivery experience from the onset of labour pains until delivery. Structured questions were then asked about the home-based birth attendant, materials used during delivery, any interventions or practices that occurred during the delivery, specific management of the retained placenta, and information on emergency transfers to a health facility. The nutritional status was assessed by calculating body mass index (BMI=weight (kg) ÷ height (m)^2^) from measures taken at seven days postpartum. Undernutrition was defined as BMI <18.5 at approximately 27 weeks of pregnancy (17). Trained female physicians assessed anaemia by examining conjunctiva at 14-22 days postpartum. The birth attendant assessed malposition of the infant during labour. Operational definitions for delivery-related complications and postpartum morbidity were developed based on the literature. These operational definitions synthesized maternal reports of signs and symptoms into specific complications and morbidities and were associated in expected ways with concurrent illness-events and medically-expected outcomes. Methods for classifying complications and morbidity into operational definitions have been described elsewhere (2).

Of 1,506 women, 1,235 (82%) gave birth at home. Of 627 home-based birth attendants (*dais*) identified by the study women, 489 (89%) were interviewed by trained nurses using a structured questionnaire with both open-ended questions and prompted responses to gather information on their training and experience. There were no significant differences in the percentage of women reporting complications or postpartum morbidity for women whose birth attendants were interviewed and those whose birth attendants were not interviewed. Of 138 *dais* not interviewed, 15% lived outside Dhaka, 83% could not be traced out, 1.5% refused interviews, and 1.5% died prior to the interview.

A checklist was used for assessing services, equipment and supplies, general delivery practices, and admission criteria for each of the 20 facilities where study women gave birth. Another checklist was used for collecting information from maternal clinical records on complications and treatments used for each study woman who delivered in a facility. Medical notes were located for 240 (89%) of 268 deliveries at facilities.

Johns Hopkins Committee on Human Research and Ethical Review Committee of ICDDR,B granted ethical approval for the study. All women gave informed consent prior to their enrollment in the study.

Quality of data was ensured through continuous field supervision and re-interviews of approximately 15% (n=225) of the 1,506 respondents for selected questions. Two persons independently coded open-ended questions. Disagreements were discussed and consensus reached. For *dais* with missing interview data, the median days of training and experience category were assigned based on the mother's description of her *dai* being ‘trained’, ‘untrained’, or ‘not normally doing deliveries’. Economic status was based on possession of household items and categorized as low (0-2 item(s)), medium (3-4 items), or high (5-6 items) (Cronbach's alpha=0.74) (18). Women who reported receiving advice on nutrition during pregnancy and a physical examination, including palpation of abdomen, checking for pedal oedema, and measuring blood pressure, were categorized as having received antenatal care. Delivery practices were identified from open-ended responses mothers gave regarding procedures and treatments they received during labour and delivery. There were prompts in the questionnaire for treatments to remove the placenta. Postpartum morbidity assessed within 14-22 days after delivery included at least one of the following conditions: perineal tears; weakness or pain in the leg; pelvic infection; urinary tract infection; vaginal tract infection; secondary postpartum bleeding; uterine prolapse; fistula; and/or feeling poorly with no specific morbid condition. Female physicians assessed these conditions which were categorized using operational definitions (2).

Data were analyzed using the SPSS/PC+DOS version 4.0 (19). Factors associated with postpartum morbidity for births at home and facility were examined in relation to maternal reports of birthing practices. Numbers of emergency deliveries (n=92) were too small for statistical testing after controlling for possible confounding factors.

Pearson's chi-square test was used for testing for associations among maternal characteristics, delivery-related complications, maternal reports of birthing practices, and postpartum morbidity by delivery-location (home, elective facility, or emergency transfer to facility). The associations between traditional birth attendants’ reports of delivery practices and level of experience were also tested using Pearson's chi-square test. Logistic regression was used for determining significant associations with postpartum morbidity controlling for maternal characteristics and delivery-related complications. Variables were selected for the logistic regression models using backward-stepwise selection; variables significant at the 0.20 level were included in the final model. Model fit was assessed using the chi-square goodness-of-fit statistic and the proportion of positives and negatives (20).

## RESULTS

### Sociodemographic characteristics

The study women (n=1,506) lived in slum areas of Dhaka under crowded conditions, with 81% living in a one-room dwelling, 86% using a shared community water source, and 94% using shared community latrines. Education levels were low, with 75% of women having no formal education. Most (94%) women were Muslim, and the remaining 6% were primarily Hindu. As defined through maternal reporting and operational definitions, 75% of the study women were classified as having postpartum morbidity, and 36% as having serious complications during delivery (2). Most (97%) women had livebirths, and there were two maternal deaths. One death occurred during delivery at home (possibly due to sepsis), while the other one was an emergency transfer to a facility (probable eclamptic convulsions).

Most (82%) deliveries were at home, with an additional 6% beginning labour at home but transferred to a health facility prior to delivery (emergency transfer) and 12% going to a facility prior to active labour (delivery at elective facility). Four deliveries at home were transferred to facilities immediately postpartum (two for retained placenta and two for newborn-related complications). About one-fourth of women who made emergency transfers were referred between two or more facilities.

Women who delivered in facilities (both elective and emergency transfers) were of significantly higher economic status, were better educated, and were significantly more likely to have received antenatal care than women who gave birth at home. There were no significant differences in parity, nutritional status, anaemia, or serious delivery-related complications between those who delivered at home and who delivered at elected facility. Postpartum morbidity was not significantly different by delivery-location (p=0.58) (Table 1). Emergency transfers, however, were more likely than deliveries at home or at elective facility among primipara (odds ratio [OR]=1.9; p<0.01,) and among those who met the operational definitions for serious delivery-related complications (OR=3.4; p<0.01) (data not shown).

### Maternal reports of childbirth practices

Practices of birth attendants were reported by women at the interview 72 hours postpartum. Practices to force gagging to remove the placenta, using a knee or other objects to apply manual pressure to the abdomen, and delivering on a mud or straw surface were significantly associated with home-based birth attendants. Among women where forced gagging was employed to remove the placenta, the most common methods were to put fingers or hair down the throat (48%) or for the woman to drink kerosene (2%). Facility-based birth attendants were significantly more likely to apply hard manual pressure to the abdomen, wash their hands with disinfectant or soap prior to the delivery, and use labour-augmenting drugs than home-based birth attendants. Repeated vaginal examinations were common for all birth attendants (61% of births at home, 87% of deliveries at elective facility, and 88% of emergency transfers to facilities) (Table 1).

**Table 1:** Proportion of women (n=1,506) by maternal characteristics, reported delivery practices, and delivery-location

Characteristics	Percentage of women			
	Delivery at home (n=1238)	Delivery at elective facility (n=180)	Emergency transfer to facility (n=88)†	p value∗

Maternal characteristics				
Primipara	24	26	39	<0.01
Grand-multipara (5+ births)	22	24	17	0.43
Undernourished	13	11	8	0.25
Severe anaemia	7	6	4	0.51
Economic status				
Lowest (0-1 asset(s))	26	8	10	
Low (2-4 assets)	56	63	57	
Moderate (5-6 assets)	18	29	33	<0.01
Maternal education (years)				
None	76	66	72	
1-6	21	23	17	
7+	3	11	12	<0.01
Use of antenatal care	29	84	64	<0.01
Reported serious complications of labour/delivery‡	35	37	55	<0.01
Delivery practice				
Deliver on mud/straw foor	72	0	1	<0.01
Hand-washing with disinfectant or soap	62	100	67	<0.01
Multiple vaginal examinations	61	87	88	<0.01
Forced gagging to remove placenta	51	1	5	<0.01
Hard manual pressure on abdomen	21	44	28	<0.01
Labour-augmenting medicines	19	40	69	<0.01
Manual removal of placenta	9	17	14	<0.01
Pressure with knee/object on abdomen	6	0	3	<0.01
Pull on umbilical cord	10	4	5	<0.01
Internal version	2	1	3	0.24
Postpartum morbidity	75	72	75	0.58

∗Chi-square test for no association between women with indicated variable by place of delivery;

†Refers to reported events which may have occurred during attempted delivery at home, prior to transfer;

‡Includes premature rupture of membranes, prolonged labour, retained placenta, hypertensive disorders of pregnancy, convulsions, and intrapartum bleeding

### Training and experience of birth attendants

All facility-based delivery attendants were either trained nurses or doctors. Of 1,238 births at home, 98% (n=1,213) were attended by *dais*. The mean age of *dais* (n=489) was 46 years, and almost none had formal education. Sixteen percent (n=78) had formal training or clinic/hospital work experience, while the large majority (75%) had no formal training but had some practical experience. Of the *dais* with no formal training, over 70% had conducted 50 or more deliveries, and 58% had conducted 100 or more deliveries in their lifetime, whereas 88% of those with formal training had conducted 100 or more deliveries (data not shown).

Birthing practices varied with level of formal training and with practical experience. *Dais* (traditional birth attendants) with formal training were significantly more likely to force gagging to remove the placenta and to use injectable oxytocin to augment labour (data not shown). *Dais* with more experience were significantly more likely to report washing hands with disinfectant or soap prior to delivery, conducting vaginal examination(s) during labour and the postpartum, changing the position of the baby in-utero, and using injectable oxytocin to augment labour (Table 2).

**Table 2:** Percentage of traditional birth attendants (n=489) reporting delivery practices stratified by reported experience

	Number of deliveries conducted in lifetime					
Delivery practice	<10 (n=68)	10-49 (n=56)	50-99 (n=57)	100-499 (n=108)	500+ (n=200)	p value∗

Hand-washing with disinfectant or soap prior to delivery	28	41	39	45	54	<0.01
Vaginal examination(s) during labour	37	68	*77*	69	81	<0.01
Vaginal examination postpartum†	9	20	*27*	22	34	<0.01
Internal version‡	0	14	23	15	24	<0.01
Use of injectable oxytocin to augment labour	7	21	47	42	52	<0.01

∗Chi-square test for no association between practice and number of deliveries conducted in lifetime;

†Most often reported to manually remove placenta;

‡Includes descriptions of ‘reaching inside to pull baby out’ and ‘reached inside to change position’

### Factors associated with postpartum morbidity among births at home

Among women with deliveries at home, self-reported postpartum morbidity was significantly associated with risk parity (primiparity or grand-multiparity of five or more births), poorest economic strata, undernutrition, anaemia, meeting operational definitions for delivery-related complications, and malposition of the infant. With regard to delivery practices, postpartum morbidity was significantly associated with having two or more vaginal examinations and reports of practices to expel the placenta or stop bleeding (forced gagging, applying hard manual pressure to the abdomen, or pulling on the umbilical cord) (Table 3). Morbidity was not associated with manual removal of the placenta or with use of labour-augmenting drugs.

**Table 3:** Proportion of women (n=1,506) with reported postpartum morbidity by maternal characteristics, reported delivery practices, and delivery-location

	Percent of women with postpartum morbidity		
Characteristics	Delivery at home (n=1,238)	Delivery at elective facility (n=180)	Emergency transfer to facility (n=88)

Maternal characteristics			
Risk parity	78†	76	66∗
Undernourished	82†	85	88
Poorest economic strata	80†	76	97†
Anaemia	85†	80	na
Malpositioned infant	92†	na	na
Any reported delivery-related complications	83†	87†	71
Use of antenatal care	79	70	65
Delivery practice			
*Dai* wash hands with soap	74	‡	‡
Forced gagging to remove placenta	78§	na	na
Hard manual pressure on abdomen	80§	75	81
Pressure with knee/object to abdomen	78	na	na
Pull umbilical cord	83†	86	75
Manual removal of placenta	82	94	69
Internal version	79	na	na
Labour-augmenting medicines	76	71	73
Multiple vaginal examinations	78†	73	74

na=Sample less than 6 women

∗p<0.05 for lower morbidity associated with characteristics for location of delivery;

†p<0.05 for higher morbidity associated with characteristics for location of delivery;

‡Not collected for deliveries at facility since respondent would not necessarily know this information;

§p<0.10 for higher morbidity associated with characteristics for location of delivery

In the logistic regression model, risk parity, poverty, and undernutrition were significantly associated with postpartum morbidity. Women who met the operational definitions for delivery-related complications and those reporting the child was malpositioned during labour were also significantly more likely to report postpartum morbidity. After controlling for maternal characteristics and delivery-related complications, multiple manual vaginal examinations were significantly associated with postpartum morbidity (OR=1.5; p=0.01). Maternal age, anaemia, and delivery practices to expel the placenta (hard manual pressure to the abdomen and pulling on umbilical cord) were not significantly associated with postpartum morbidity. Morbidity was higher for births at home among women who had received antenatal care, although this was not statistically significant (p=0.05) (Table 4).

**Table 4:** Logistic regression model for variables associated with postpartum morbidity for deliveries at home (n=1,234)

Variable	Adjusted odds ratio	95% CI	p value

Maternal characteristics			
Risk parity	1.4	1.04-1.80	0.02
Maternal age (years)	1.0	1.00-1.04	0.89
Poorest strata	1.7	1.28-2.23	0.00
Undernourished	1.6	1.05-2.52	0.03
Anaemia	1.7	0.88-3.09	0.12
Use of antenatal care	1.4	1.00-1.87	0.05
Malpositioned infant	3.3	1.17-9.53	0.02
Any reported delivery-related complications	1.7	1.27-2.31	0.00
Delivery practices			
Hard pressure to abdomen with hand or object or pull umbilical cord	1.3	0.97-1.78	0.08
Multiple vaginal exam	1.5	1.12-1.96	0.01

CI=Confidence interval

### Factors associated with postpartum morbidity among deliveries at elective facility

Among women with deliveries at elective facilities, postpartum morbidity was significantly associated with intrapartum bleeding and other delivery-related complications, multiple manual vaginal examinations, use of antenatal care during pregnancy, and the type of facility (data not shown). After adjusting for confounding variables, postpartum morbidity was not significantly associated with maternal characteristics, such as grand-multipara, age, nutritional status, education, or with episiotomy. Morbidity varied by facility. Women who gave birth at non-governmental organization or government clinics were 2.2 times more likely to report postpartum morbidity compared to women who gave birth at government maternity hospitals (p<0.01), while women who gave birth at private facilities were less likely to report postpartum morbidity (OR=0.52; p=0.06). Women who received antenatal care were less likely to report postpartum morbidity (OR=0.26; p=0.03), while women who met the operational definitions for intrapartum bleeding or other delivery-related complications were 2.8 and 2.9 times more likely to report postpartum morbidity respectively. While a large proportion of women reported the practice of applying hard pressure to the abdomen during labour and postpartum, this practice was not significantly associated with morbidity in the multivariate analysis. Women with multiple manual vaginal examinations were 2.4 times more likely to report postpartum morbidity, a strong although not statistically significant association (p=0.09; data not shown).

## DISCUSSION

Despite the availability of health facilities in Dhaka, Bangladesh, a large proportion of women deliver at home and report delivery-related complications and subsequent postpartum morbidity (2). Most deliveries at home in slum areas are conducted by women with some practical experience but with little formal training. Although postpartum morbidity was not significantly different among women with home- or facility-based births, harmful home-based delivery practices, such as multiple vaginal examinations and use of injectable oxytocic medications to augment labour, were commonly used by more experienced and trained *dais*.

Few studies have measured birthing practices and their effects on postpartum morbidity. In this study, women with deliveries at home were significantly more likely to report traditional practices to remove the placenta (forced gagging, hard manual pressure on the abdomen, and pulling the umbilical cord) than women who gave birth at health facilities. Some birthing practices for deliveries at home documented by this study, such as pulling on the umbilical cord (10%) and applying hard manual pressure on the abdomen (21%), were not independently significantly associated with postpartum morbidity in the multivariate analyses, due to small numbers. However, when combined, these practices were associated with morbidity (p=0.08) for deliveries at home but not for deliveries at elective facility. If the provider is unskilled, it is likely that pressure and traction on the umbilical cord will be conducted incorrectly. Practices, such as applying pressure with knee/object on the abdomen (6%) and being forced to gag to remove the placenta (51%), are never safe.

Multiple vaginal examinations were prevalent in all deliveries (both at home and facility). After controlling for maternal characteristics and delivery-related complications, this practice was significantly associated with postpartum morbidity among women with home at births (p=0.01) and among women with deliveries at facility (p=0.09). Goodburn *et al.* reported a similar finding in a study conducted in rural Bangladesh (16). Almost half (48%) of traditional birth attendants inserted their hands into the woman's vagina during delivery which was significantly associated with infection at two weeks postpartum controlling for confounding factors. In this study, discussions with *dais* and women's narratives indicated that examinations are frequently conducted to determine the position of the infant and the progression of labour. These multiple examinations were the one procedure noted to be part of the normal delivery practice both at home and at facility. This finding indicates that multiple vaginal examinations need to be discouraged while promoting non-invasive means, such as abdominal palpation, of assessing infant position and labour progress for all levels of delivery providers.

The use of labour-augmenting drugs to facilitate labour and delivery was reported by 19% of women with births at home with experienced *dais* significantly more likely to use these drugs than less-experienced *dais*. Although there was no indication of increased postpartum morbidity or adverse outcomes among women who received labour-augmenting medications, this practice is potentially dangerous when administered by untrained persons at home (21). The use of oxytocics and other invasive treatments by more experienced *dais* requires further study.

Women who delivered at home were more likely to be of lower economic status, higher maternal age, and risk parity (primiparity or grand-multiparity of five or more births) compared to women with births at elective facility. These findings are similar to those of other studies in the maternal health literature (22). Despite these differences in maternal characteristics, postpartum morbidity did not differ significantly by birth-location. One possible explanation is that last-minute complications and/or anxiety prompted women to electively give birth at a facility. Over 30% of women with births at facility reported that they chose this location because of poor health or fear of complication, and 9% of these women reported premature rupture of membranes. These complications may have influenced their decision to go to a facility rather than attempt delivery at home.

There were several strengths of and limitations to this study. The strengths include the large sample size and the detailed information collected from the study women and the delivery providers/delivery institutions on common birth practices by the providers and specific birth practices experienced by each study woman. The results of the study could be limited by several factors. First, the study relied on self-reports of complications and postpartum morbidity. Although self-reports over- or underestimate medically-defined complications and/or morbidity (23), internal consistency between self-reported symptoms as classified by operational definitions and medically-expected outcomes has been demonstrated (2).

Another limitation is the lack of information on interventions that occurred prior to the woman's arrival at the facility and her state of health upon arrival. Women with deliveries at elective facility were sometimes examined by a *dai* who confirmed the necessity of transferring to a facility. The lack of information on the state of health prior to labour and interventions that occurred at home limit the ability to make definitive statements regarding facility-based delivery practices and postpartum morbidity. It is also uncertain whether *dais* or the women and their families were the main factors influencing the use of facilities for delivery-related complications. *Dais* may be handling complications and employing practices beyond their skill level in attempts to help woman who refuse facility-based assistance.

It is evident from this study that women suffer needlessly from delivery-related complications and postpartum morbidity in urban slum areas of Bangladesh. To improve maternal morbidity and mortality, safe motherhood programmes must develop effective strategies to discourage potentially-harmful home-based delivery practices demonstrated to contribute to morbidity and to more closely examine the factors that prompt women to use facility-based care.
